# Ubiquitin-conjugating enzyme E2T promotes tumor stem cell characteristics and migration of cervical cancer cells by regulating the GRP78/FAK pathway

**DOI:** 10.1515/biol-2021-0108

**Published:** 2021-10-01

**Authors:** YanMei Liu, WenLi Ji, Na Yue, Weidong Zhou

**Affiliations:** Department of Pathology, The 3rd Affiliated Teaching Hospital of Xinjiang Medical University (Affiliated Cancer Hospital), Urumqi City, Xinjiang Uygur Autonomous Region, 830000, China; Department of Obstetrics and Gynecology, The First Hospital of Yulin, No. 59 Wenhua Road, Suide County, Yulin City, Shanxi Province, 718000, China

**Keywords:** UBE2T, stem cell, cervical cancer, progression, GRP 78, FAK

## Abstract

Ubiquitin-conjugating enzyme E2T (UBE2T) functions as an E2 ubiquitin-conjugating enzyme in the ubiquitin-proteasome degradation system and mediates cellular processes, such as cell cycle, proliferation, and differentiation. UBE2T has been considered to be an oncogene in a variety of tumors. However, the oncogenic role of UBE2T in cervical cancer remains unclear. In this study, our results first showed that the expression of UBE2T was higher in both of cervical cancer tissues and cells than that in the normal tissues and cells. Knockdown of UBE2T reduced cervical cancer cell viability and suppressed the proliferation, invasion, and migration. However, overexpression of UBE2T contributed to cervical cancer cell growth and metastasis. Moreover, UBE2T overexpression cervical cancer cells demonstrated enhanced self-renewal capacity with upregulation of SOX2, Oct-4, and Nanog protein. Silencing of UBE2T downregulated protein expression of SOX2, Oct-4, and Nanog in cervical cancer cells reduced self-renewal capacity. Furthermore, ectopic UBE2T expression promoted protein expression of glucose-regulated protein 78 (GRP78) and focal adhesion kinase (FAK) phosphorylation in cervical cancer cells. The knockdown of UBE2T reduced protein expression of GRP78 and FAK phosphorylation. Collectively, UBE2T promoted cervical cancer stem cell traits and exerted an oncogenic role through activation of the GRP78/FAK pathway.

## 1 Introduction

Cervical cancer ranks as the fourth leading cause of malignance-related mortality in women worldwide and the third most common cancer [1,2]. Although the widespread implementation of screening programs has reduced morbidity and mortality of cervical cancer in recent years, it remains a major public health problem, particularly in advanced cases [3]. Recent advancements in therapeutic strategies, such as chemotherapy, surgery, and radiotherapy, have improved patients’ survival rate with primary cervical carcinoma [4]. However, the advanced cases of cervical cancer with local or distant metastasis reduce the 5-year survival rate to 50% [5]. Therefore, efforts that focus on the identification of tumor-specific markers that predict the biological behavior of cervical cancer would be crucial for the prevention of cervical cancer cell motility and aggressiveness, further repressing the cancer occurrence and development.

Ubiquitin-conjugating enzyme E2T (UBE2T) belongs to the ubiquitin-proteasome system that covalently links ubiquitin to the lysine residues of target proteins and chains [6]. Since polyubiquitin chains are known to play a key role in the regulation of tumor progression, UBE2T is regarded as a potential therapeutic target for tumors [6]. Study has shown that UBE2T was upregulated in bladder cancer tissues and cell lines, and knockdown of UBE2T reduced the proliferation of bladder cancer cells, induced cell cycle arrest in the G2/M phase, and increased the apoptosis [7]. UBE2T has also been shown to be an oncogene in non-small cell lung cancer [8], renal cell carcinoma [9], and hepatocellular carcinoma [10]. However, the role of UBE2T in cervical cancer is still not clear.

Glucose-regulated protein 78 (GRP78) is expressed in the lung, brain, or liver and functions as a stress-induced endoplasmic reticulum chaperone [11]. Overexpression of GRP78 in various tumors was found to be implicated in biological processes, including cell survival, invasion, and metastasis [11]. Focal adhesion kinase (FAK) is a nonreceptor tyrosine kinase that plays a key role in modulation of integrin-mediated signaling pathways between cells and the extracellular matrix [12]. FAK functions as a critical regulator in tumor cell survival, invasion, and metastasis [12]. GRP78 has been shown to promote tumor progression through activation of FAK pathway [13]. GRP78/FAK was also reported to be involved in the tumor metastasis of cervical cancer [14]. Downregulation of GRP78 was implicated in the suppression of cervical cancer development [15]. Since previous study has shown that UBE2T interacted with GRP78 and promoted glioma cell migration and invasion [16], we hypothesized that UBE2T may function as an oncogene in cervical cancer through GRP78/FAK pathway in this study.

## 2 Materials and methods

### 2.1 Clinical specimens

Forty pairs of cervical cancer and adjacent normal tissues were obtained from patients which were pathologically diagnosed as cervical cancer. Patients with written informed consents were recruited at Affiliated Cancer Hospital of Xinjiang Medical University from 2016 to 2019.

**Informed consent:** Informed consent has been obtained from all individuals included in this study.**Ethical approval:** The research related to human use has been complied with all the relevant national regulations, institutional policies, and in accordance with the tenets of the Helsinki Declaration and has been approved by the Affiliated Cancer Hospital of Xinjiang Medical University.

### 2.2 qRT-PCR

RNAs were isolated from cervical cancer and normal tissues via Trizol (Invitrogen, Carlsbad, CA, USA). The RNAs were reverse-transcribed into cDNAs and conducted with qRT-PCR analysis of UBE2T using SYBR Green Master (Roche, Mannheim, Germany). The following primers for UBE2T (forward: 5′-TTGATTCTGCTGGAAGGATTTG-3′; reverse: 5′-CAGTTGCGATGTTGAGGGAT-3′) and GAPDH (forward: 5′-TGACCACAGTGGATGCCAT-3′; reverse: 5′-TTACTCCTTGGAGGCCATGT-3′) were used in this study. GAPDH was used as endogenous control.

### 2.3 Cell culture and transfection

Human cervical cancer cell lines (HeLa, Caski, Siha) and normal human endocervical epithelial cell line (Endl/E6E7) were obtained from Cell Resource Center of Shanghai Academy of Sciences (Shanghai, China). Cells were cultured in Dulbecco’s modified Eagle’s medium (GE Healthcare Life Sciences, Little Chalfont, UK) containing 10% fetal bovine serum (GE Healthcare Life Sciences) at a 37°C incubator. For cell transfection, siRNA-mediated knockdown (si-UBE2T), pcDNA-mediated overexpression of UBE2T (pcDNA–UBE2T), and pcDNA-GRP78 were provided by GenePharma (Suzhou, China). HeLa and Caski were seeded into a 6-well plate and transfected with siRNAs and pcDNAs by Lipofectamine 2000 (Invitrogen).

### 2.4 Western blot

RIPA buffer (Ding Guo Chang Sheng Biotech, Beijing, China) was used to lyse the tumor tissues and cells on ice. The protein concentration of the supernatants collected from the lysates was calculated using a bicinchoninic acid protein assay kit (Pierce Biotechnology, Rockford, IL, USA). Samples (30 µg) were separated by SDS-PAGE and then transferred to PVDF membrane. The membrane was probed with anti-UBE2T and anti-GAPDH (1:2,000; Abcam, Cambridge, MA, USA), anti-FAK and anti-p-FAK (1:2,500; Abcam), anti-SOX2 and anti-Oct4 (1:3,000; Abcam), anti-Nanog, or anti-GRP78 (1:4,000; Abcam). Following incubation with horseradish peroxidase-conjugated immunoglobulin G (1:5,000; Abcam), the blots were detected by enhanced chemiluminescence (KeyGen, Nanjin, China).

### 2.5 Cell viability and proliferation assays

HeLa and Caski under siRNAs or pcDNAs transfection were seeded into a 96-well plate for 24, 48, or 72 hours. MTT solution (5 mg/mL; Beyotime, Beijing, China) was added into each well and incubated for 4 h. Following dissolving of the formazan products by DMSO, absorbance at 490 nm was detected by a microplate reader (Bio-Rad, Hercules, CA, USA). For cell proliferation assay, HeLa and Caski under siRNAs or pcDNAs transfection were seeded in a 6-well plate and cultured for 14 days. Paraformaldehyde-fixed and crystal violet-stained cells were observed under a light microscope (Olympus, Tokyo, Japan).

### 2.6 Wound healing and invasion assays

For wound healing, HeLa and Caski cells under siRNAs or pcDNAs transfection were seeded into a six-well plate until 90% confluence. A pipette tip was used to induce a scratching wound in the well. Twenty-four hours later, the wound width was calculated under an inverted Olympus IX50 microscope. For invasion assay, HeLa and Caski cells in serum-free medium were placed into the upper chamber of Matrigel-coated Transwell chamber (Biosciences, San Jose, CA, USA). The lower chamber was supplied with medium containing 20% fetal bovine serum. After 24 h of incubation, cells in the lower chamber were fixed in 4% paraformaldehyde and stained with hematoxylin. Cells were counted under the light microscope.

### 2.7 Tumorsphere culture

HeLa and Caski under siRNAs or pcDNAs transfection were cultured in medium (DMEM/F12 basal medium containing 20 ng/mL basic fibroblastic growth factor, 20 ng/mL human recombinant epidermal growth factor, N2 and B27 supplements) (PeproTech Inc., Rocky Hill, NJ, USA) and seeded into a 24-well ultralow attachment plates at a density of 200 cells per well. Two weeks later, the tumorspheres were aroused and counted under the light microscope.

### 2.8 Statistical analysis

Results from three independent experiments were presented as mean ± SD. Statistical analyses between different groups were performed with one-way analysis of variance or Student’s *t* test under SPSS19.0 software. Values were considered significant at *p* < 0.05.

## 3 Results

### 3.1 Upregulation of UBE2T in cervical cancer

**Figure 1:**
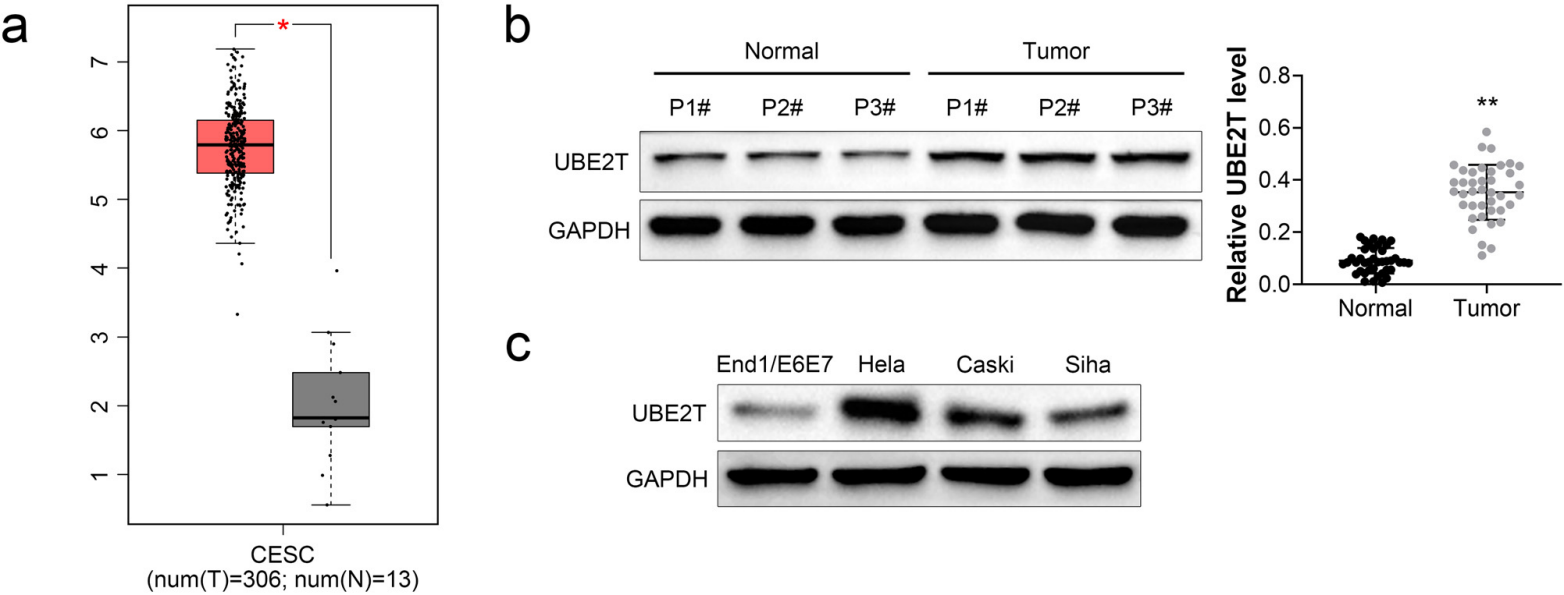
Upregulation of UBE2T in cervical cancer. (a) UBE2T was upregulated in the cervical cancer tissues (*n* = 306) compared to the normal tissues (*n* = 13) based on GEPIA database shown. (b) Protein and mRNA expression of UBE2T were upregulated in the cervical cancer tissues compared to the normal tissues. (c) Protein expression of UBE2T was upregulated in the cervical cancer cells (HeLa, Caski, and Siha) compared to the normal cells (End1/E6E7).

**Figure 2:**
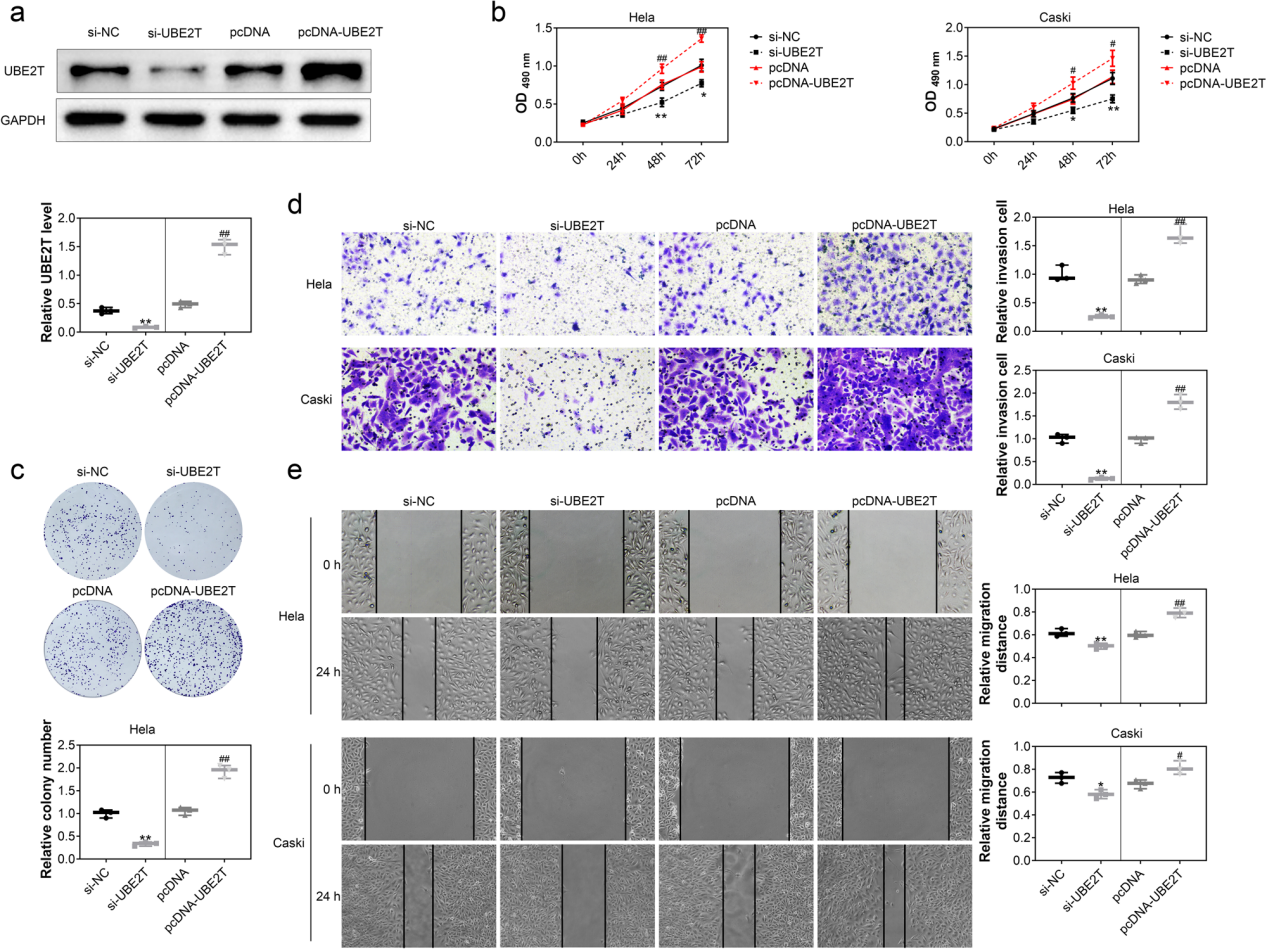
UBE2T contributed to cervical cancer cell growth. (a) Transfection with si-UBE2T in HeLa cells decreased the protein expression of UBE2T, while pcDNA–UBE2T increased the expression. (b) Cell viability of HeLa and Caski was reduced by si-UBE2T, while enhanced by pcDNA–UBE2T. (c) Knockdown of UBE2T repressed the cell proliferation of HeLa and Caski, while overexpression of UBE2T promoted the proliferation. (d) Knockdown of UBE2T repressed the cell invasion of HeLa and Caski, while overexpression of UBE2T promoted the invasion. (e) Knockdown of UBE2T repressed the cell migration of HeLa and Caski, while overexpression of UBE2T promoted the migration. *, ** vs siNC, *p* < 0.05, *p* < 0.01. ## vs pcDNA, *p* < 0.01.

Microarray analysis based on GEPIA (Gene Expression Profiling Interactive Analysis) database showed that UBE2T was upregulated in the cervical cancer tissues (*n* = 306) compared to the normal tissues (*n* = 13) (Figure 1a). Western blotting assay also identified the upregulation of UBE2T in the tumor tissues (Figure 1b) and cervical cancer cell lines (Figure 1c). Moreover, mRNA expression of UBE2T was also upregulated in the cervical cancer tissues compared with normal tissues (Figure 2b). These results suggested that UBE2T might be related to cervical cancer progression.

### 3.2 UBE2T contributed to cervical cancer cell growth

HeLa and Caski were conducted with gain- and loss-of-functional assays through transfection with siRNA and pcDNA, respectively. Transfection with si-UBE2T decreased the protein expression of UBE2T, while pcDNA–UBE2T increased its expression (Figure 2a). Cell viability of HeLa and Caski was reduced by si-UBE2T, while enhanced by pcDNA–UBE2T (Figure 2b). Moreover, knockdown of UBE2T repressed the cell proliferation (Figure 2c), invasion (Figure 2d), and migration (Figure 2e) of HeLa and Caski. However, the cell proliferation (Figure 2c), invasion (Figure 2d), and migration (Figure 2e) of HeLa and Caski were promoted by overexpression of UBE2T. These results indicated the proliferative and pro-invasive effects of UBE2T on cervical cancer.

### 3.3 UBE2T contributed to stemness of cervical cancer cell

**Figure 3:**
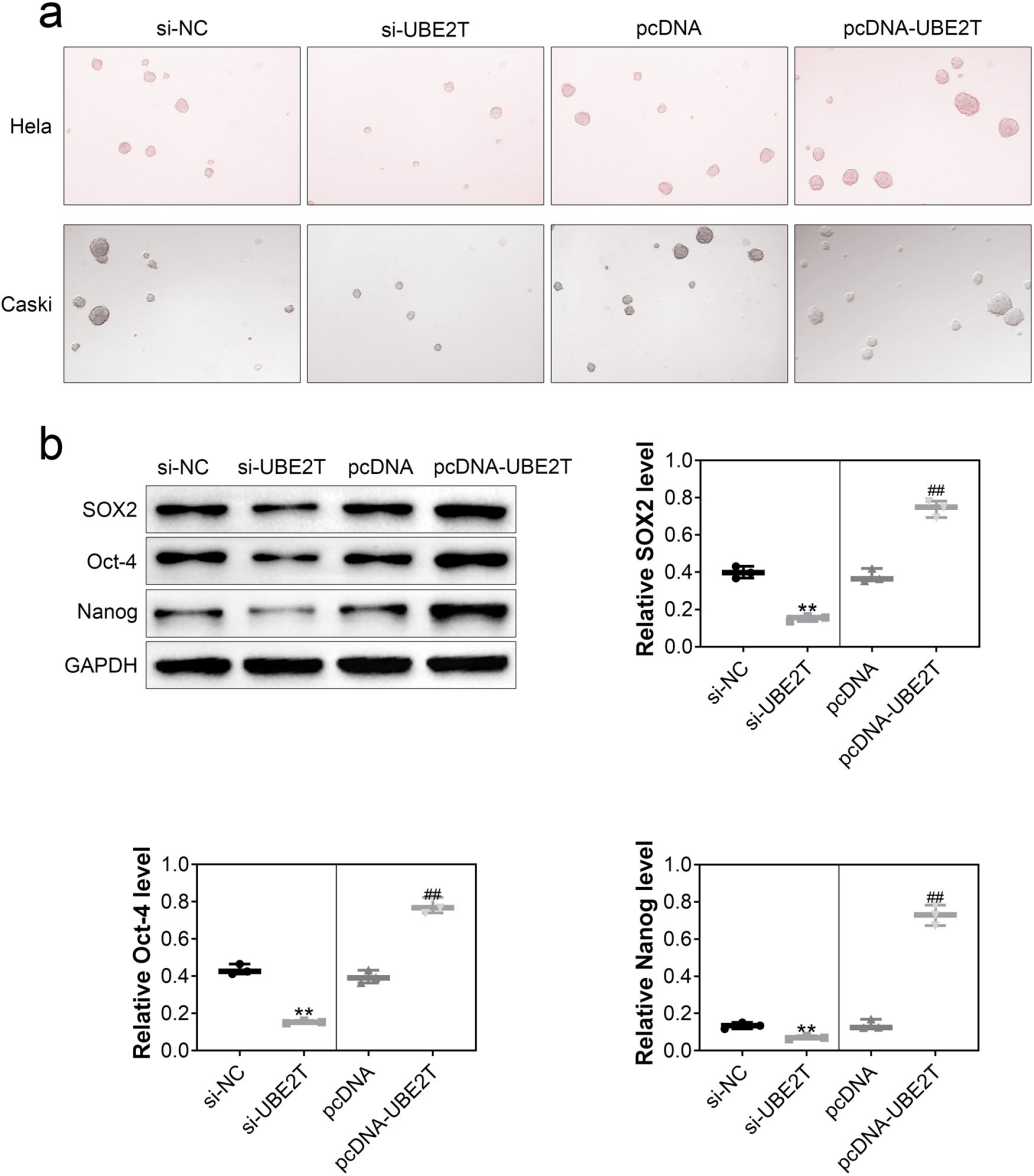
UBE2T contributed to stemness of cervical cancer cell. (a) UBE2T-silenced HeLa and Caski cells showed reduced typical tumorspheres compared to the control, while overexpression of UBE2T enhanced the tumorspheres. (b) Knockdown of UBE2T decreased protein expression of SOX2, Oct4, and Nanog in HeLa cells, while overexpression of UBE2T increased the protein expression. ** vs siNC, *p* < 0.01. ## vs pcDNA, *p* < 0.01.

The effect of UBE2T on self-renewal capacity of cervical cancer cell was assessed through tumorsphere formation assay. UBE2T-silenced HeLa and Caski cells showed reduced typical tumorspheres compared with the control (Figure 3a), while overexpression of UBE2T enhanced the tumorspheres (Figure 3a). The stem cell-related transcription factors, including SOX2, Oct4, and Nanog, were decreased in UBE2T-silenced cells (Figure 3b) and increased in UBE2T-overexpressed cells (Figure 3b), revealing that cervical cancer stem cell traits were promoted by UBE2T.

### 3.4 UBE2T contributed to GRP78/FAK activation

**Figure 4:**
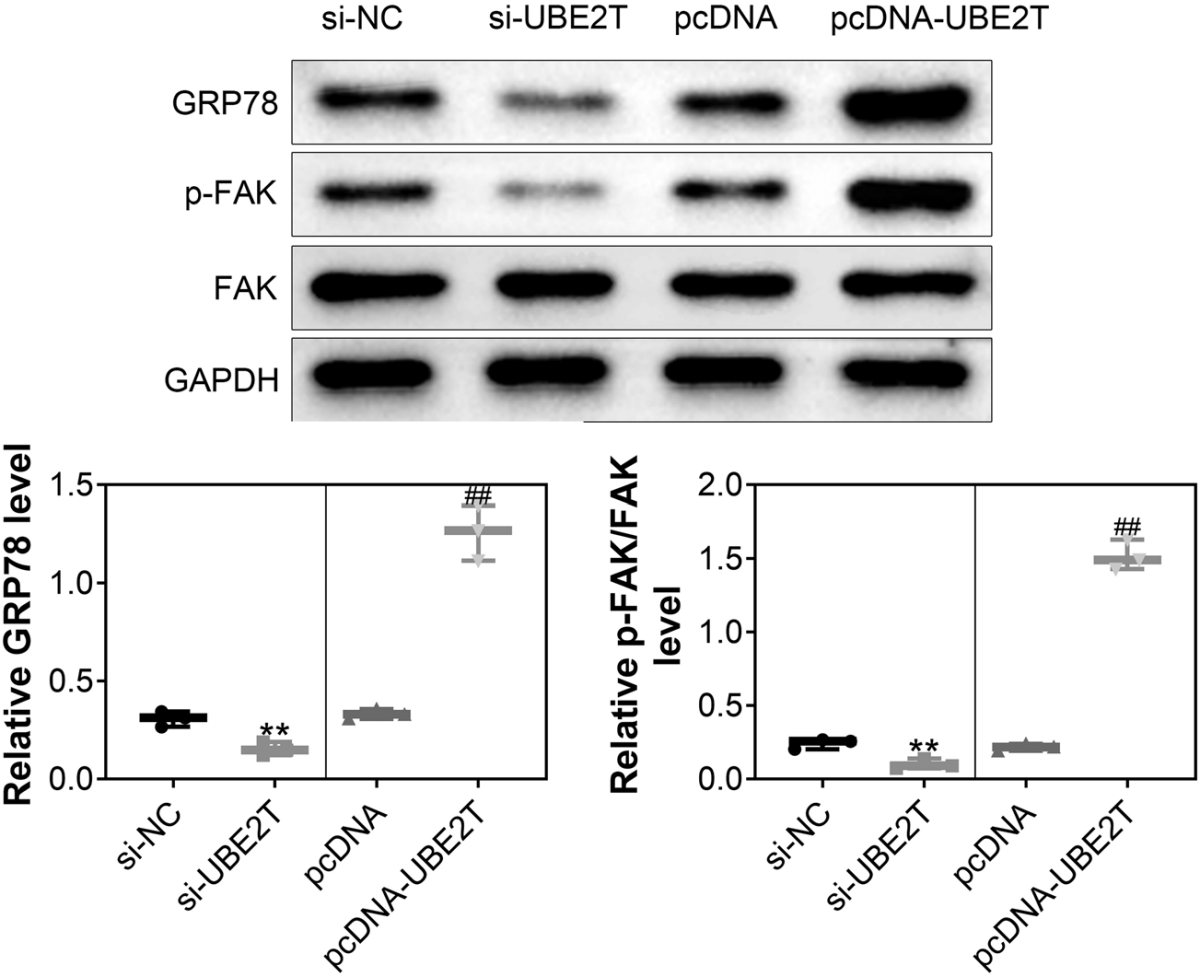
UBE2T contributed to GRP78/FAK activation. Knockdown of UBE2T decreased protein expression of FAK phosphorylation and GRP78 in HeLa cells, while overexpression of UBE2T increased the protein expression. ** vs siNC, *p* < 0.01. ## vs pcDNA, *p* < 0.01.

**Figure A1:**
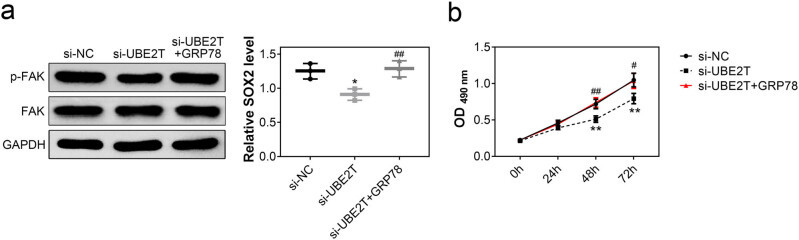
Overexpression of GRP78 attenuated UBE2T silence-induced decrease of p-FAK. (a) Decrease of cell viability in HeLa induced by silence of UBE2T was also restored by overexpression of GRP78 (b). *, ** vs siNC, *p* < 0.05, *p* < 0.01. #, ## vs si-UBE2T, *p* < 0.05, *p* < 0.01.

Protein expression of FAK was not affected by UBE2T-silencing or UBE2T-overexpressing in HeLa cells (Figure 4). However, FAK phosphorylation was reduced in UBE2T-silenced HeLa cells and enhanced in UBE2T-overexpressing HeLa cells (Figure 4). Moreover, GRP78 was also downregulated by knockdown of UBE2T, while upregulated by overexpression of UBE2T (Figure 4). HeLa was cotransfected with si-UBE2T and pcDNA-GRP78. Overexpression of GRP78 attenuated UBE2T silencing-induced decrease in p-FAK (Figure A1). Decreased cell viability in HeLa induced by silencing of UBE2T was also restored by overexpression of GRP78 (Figure A1), demonstrating that UBE2T contributed to cervical cancer cell growth and metastasis through GRP78/FAK activation.

## 4 Discussion

Ubiquitin-mediated degradation system, including E1, E2, and E3 enzymes, was implicated in the physiologic processes of tumor cells [17]. E2 enzymes are found to be dysregulated in a variety of cancers and function as diagnostic, prognostic, or therapeutic targets of tumors through modulation of the stability of target proteins [17]. For example, E2-EPF, an ubiquitin-conjugating enzyme, was overexpressed in the cervical cancer and associated with the aggressivity and growth of tumor cells [18]. Since UBE2T has been shown to be involved in the maintenance of chromosome stability and cell proliferation, thereby indicating the oncogenic nature in various tumors [17], the role of UBE2T in cervical cancer was then investigated in this study.

Higher expression of UBE2T in cervical cancer tissues and cells was first identified in this study. Previous study has shown that UBE2T was overexpressed in gastric cancer and predicted the poor prognosis [19]. The correlation between UBE2T and the clinical characteristics of patients with cervical cancer should be investigated to validate the diagnostic or prognostic roles in the future.

The oncogenic role of UBE2T in cervical cancer was then confirmed by the functions assays in this study. Results showed that ectopic expression of UBE2T enhanced the cell viability, cell proliferation, invasion, and migration of HeLa cells. Moreover, the cervical cancer cell growth and metastasis were suppressed by knockdown of UBE2T. Cervical cancer cells gain the mesenchymal phenotype and lose the epithelial characteristic features during tumor progression [20], and UBE2T promoted the ubiquitination-mediated FOXO1 degradation to facilitate the epithelial-mesenchymal transition of non-small cell lung cancer [21]. The effect of UBE2T on epithelial-mesenchymal transition of cervical cancer cells should be investigated in further researches.

Cervical cancer stem cells have self-renewal property and could differentiate into tumor cells for the tumorigenesis, thus representing a potential therapeutic target for the treatment of cervical cancer [22]. Leucine-rich repeat-containing G-protein-coupled receptor 5 promoted the cervical cancer stem cell traits, and knockdown of leucine-rich repeat-containing G-protein-coupled receptor 5 suppressed the tumorigenicity of cervical cancer cells [23]. UBE2T is first identified in the hematopoietic stem cells and plays critical role in the maintaining of hepatocellular carcinoma stem cells [24]. The stem cell-related transcription factors, including SOX2, Oct4, and Nanog, were decreased in UBE2T-silenced HeLa cells in this study, suggesting that knockdown of UBE2T might suppress the stem cell-like features in cervical cancer.

Different pathways, including PI3K/AKT [9], wnt/β-catenin [8], and GRP78 [16], were involved in UBE2T-mediated tumor progression. Results in this study showed that protein expression of phosphorylated FAK and GRP78 was enhanced in cervical cells with UBE2T overexpression and reduced by UBE2T silencing. GRP78, upregulated in the cervical cancer [25], has been shown to activate FAK to promote the hepatocellular carcinoma progression [26]. FAK activation was involved in the tumorigenesis of cervical cancer [27]. Therefore, results in this study indicated that UBE2T contributed to cervical cancer cell growth through activation of GRP78/FAK pathway.

In summary, UBE2T promoted the growth and metastasis of cervical cancer and facilitated the maintaining of the stem cell-like features in cervical cancer. Suppression of GRP78/FAK activation was implicated in the suppressive effect of UBE2T silencing on cervical cancer. Therefore, UBE2T might be a novel target for the treatment of cervical cancer.
